# Intranasal immunization with a Bucl8-based vaccine ameliorates bacterial burden and pathological inflammation, and promotes an IgG2a/b dominant response in an outbred mouse model of *Burkholderia* infection

**DOI:** 10.3389/fimmu.2023.1177650

**Published:** 2023-07-20

**Authors:** Megan Grund, Soo Jeon Choi, Lillie Powell, Slawomir Lukomski

**Affiliations:** Department of Microbiology, Immunology and Cell Biology, School of Medicine, West Virginia University, Morgantown, WV, United States

**Keywords:** Burkholderia, melioidosis, Vaccine, intranasal, humoral immunity, Bucl8

## Abstract

*Burkholderia pseudomallei* is a gram-negative bacterium that is the etiological agent of the tropical disease melioidosis. Currently, there is no licensed vaccine for melioidosis, but numerous candidates are being tested for protective efficacy and characterization of the elicited immune response. Our lab has previously reported the immunogenicity of a Bucl8-protein-based peptide antigen, designated L1-CRM_197_ (Cross-reacting material 197). When given subcutaneously, this vaccine formulation promoted a strong Th2 (IgG1) antibody response, however immunization did not protect from death. In this study, we hypothesized that an intranasally administered L1-CRM_197_ vaccine would induce protective mucosal immunity. To evaluate vaccine efficacy, we developed a surrogate *Burkholderia* infection model that employs outbred CD-1 mice which imitates the immunogenetic diversity of humans. Mice were immunized with either L1-CRM_197_ adjuvanted with fluorinated cyclic diguanosine monophosphate (FCDG) or with FCDG-only control. These mice were then challenged intranasally with an infectious dose of a luminescent strain of *B. thailandensis* E264 two weeks post-immunization, and correlates of protection were assessed in euthanized mice on days 1, 2, 3, and 7 post-infection. Overall, intranasal vaccination, compared to subcutaneous administration, induced a stronger Th1 (IgG2a/2b) to Th2 (IgG1) antibody response and promoted anti-L1 nasal, pulmonary, and systemic IgA. Additionally, sera IgG from L1-CRM_197_-vaccinated mice recognized whole-cell *B. thailandensis* and *B. pseudomallei*, a select agent exempt strain Bp82. Vaccination ameliorated disease indicators, including luminescent signal and bacterial cell counts, weight and temperature loss, and organ weight, which negatively correlated with IgG2a antibody levels and mucosa-stimulating cytokines IL-13 and IL-9. L1-CRM_197_-vaccinated mice also had earlier resolution of inflammatory and tissue-damaging cytokines compared to the FCDG-only controls. These results suggest a balanced humoral and cell-mediated response, along with mucosa-based immunity are beneficial for protection. Future efforts should further assess mucosal cellular and humoral mechanisms of protection and test such protection, using aerosolized *B. pseudomallei* select agent strain(s).

## 1 Introduction

*B. pseudomallei* is an opportunistic human pathogen, the causative agent of melioidosis, and a member of the namesake *Burkholderia pseudomallei complex*. This complex also includes *Burkholderia mallei*, a clonal derivative of *B. pseudomallei* that is the etiological agent of glanders in equines, a non-pathogenic *B. thailandensis*, and other close relatives (1, 2). Because of its intrinsic antibiotic resistance, propensity for aerosolization, and environmental reservoirs, *B. pseudomallei* is classified as a Tier one select agent, and thus requires use of a BSL3-level select agent laboratory. Currently there is not a melioidosis or glanders vaccine.

Several vaccine formulations have been studied, including live attenuated, subunit, outer membrane vesicles, nanoparticle-based, and nucleic acid-based approaches (3). Live attenuated vaccines have been shown to be protective against *B. pseudomallei*, eliciting both humoral and cell-mediated immunity, as well as immune-memory (4–7). For example, vaccination with *B. pseudomallei* PBK001 generated partially protective IgG and IgA antibodies, evaluated *via* passive transfer and challenge (6). However, live attenuated vaccines may have increased safety concerns (particularly in immunocompromised populations) and may not be as consistently manufactured as subunit vaccines, which can also be tailored to increase antigen-specific immunity and reduce adverse reactions. The challenge is to generate a vaccine that combines the efficacy of a live attenuated vaccine with the specificity and safety profile of a subunit vaccine.

In addition to the selection of immunogenic vaccine target(s), the right adjuvant, conjugate, and route of administration can also alter the type and strength of immune response elicited. Several subunit vaccine targets that have been identified as immunogenic, such as the T6SS protein Hcp1 (8), outer membrane protein OmpW (9), capsular polysaccharide (8, 10, 11), and peroxide-detoxifier AhpC (5, 12), elicit varying degrees of protection against disease and death. Other vaccine strategies have combined the immunogenic benefits of whole cell vaccines, more specifically their polysaccharides, with antigenicity of outer membrane proteins (OMP). These vaccine targets are often selected because they are well-conserved, immunogenic, and surface-exposed, therefore increasing immune recognition. Murine models are used in vaccine research to evaluate the host’s immune response and protection against diseases, such as melioidosis. For example, C57BL/6 inbred mice have been used to represent a Th1-biased, chronic model of melioidosis due to the extended time it takes to succumb to infection, depending upon infection parameters (13, 14). In contrast, BALB/c inbred mice represent an acute, Th2-biased model, with the course of infection and resolution generally completing within a shorter timeframe. *B. thailandensis*, which is closely related to *B. pseudomallei* but has decreased virulence, is classified as a BSL-2 organism (15, 16). Previous studies have used *B. thailandensis* as a surrogate model of melioidosis (17–19), while employing inbred mice.

*Burkholderia* collagen-like protein 8 (Bucl8) is a putative OMP component of a tetrapartite efflux pump imparting fusaric acid resistance in *B. pseudomallei.* The Bucl8 protein sequence includes two tandem outer-membrane efflux domains, as well as an extended extracellular collagen-like domain (20). The OMP portion forms a characteristic periplasmic α-barrel and outer membrane-spanning β-barrel with surface-exposed loops, designated L1 and L2 (21, 22). Bucl8 is conserved across multiple *Burkholderia* species such as *B. pseudomallei* and *B. mallei* and has homologs without the extracellular collagen-like domain in *B. thailandensis* and majority of the BCC species (22). Because of this conservation and the immunogenicity of Bucl8, we have proposed a vaccine derived from the surface-exposed epitopes, including loops L1 and L2. Our prior studies have shown that synthetic peptides based on the L1 and L2 sequences, conjugated to the genetically detoxified diphtheria toxoid CRM_197_ (Cross-reacting material 197) and adjuvanted with the AddaVax (MF59-like squalene-based oil-in-water adjuvant), elicited strong antibody titers that were IgG1 dominant when administered subcutaneously (22).

The purpose of this study is to develop a mucosal melioidosis vaccine based on the Bucl8 protein and utilizing a CD-1 strain of mice intranasally challenged with *B. thailandensi*s. To assess vaccine efficacy against the primary acute infection, we established an infectious dose of *B. thailandensis* E264 constitutively expressing a *lux*- cassette in a CD-1 mouse model. We then subcutaneously or intranasally immunized mice with vaccine formulations utilizing peptide-CRM_197_ conjugates based on the surface exposed loops of Bucl8, followed by respiratory bacterial challenge. Disease parameters such as survival, weight, temperature, and bacterial burden were used to assess level of disease and protection. Systemic and mucosal antibodies were measured to assess the humoral response to the vaccine, and cell populations and cytokine levels were used to assess the cell-mediated response. In combination, these parameters of protection can be used to evaluate vaccines’ efficacy in an outbred murine model.

## 2 Material and methods

### 2.1 Bacterial strains and growth

*B. thailandensis* strain E264 (Bt E264) is a non-capsulated BSL-2 surrogate for *B. pseudomallei* that was utilized for noted bacterial challenges. A constitutively luminescent strain of Bt E264 [Bt E264-*lux* (23)], was gifted by Dr. Heath Damron (West Virginia University) and used in indicated experiments. *B. pseudomallei* strain Bp82, an avirulent Δ*purM* mutant of strain 1026b exempt from the Select Agents list, was used to assess antibody-binding. Bacteria were routinely grown in Luria broth-Miller with shaking and on Luria agar solid medium at 37°C.

### 2.2 Animal care and use

All CD-1 IGS mice (Charles River Laboratories) experiments were approved by the West Virginia University Institutional Animal Care and Use Committees (WVU-IACUC protocol 1804013711) and performed in accordance with the National Institutes of Health Guide for the Care and Use of Laboratory Animals. Initial experiments used five- to six-week-old female and male CD-1 mice for the subcutaneous immunization study. Female CD-1 mice were selected for later studies due to their increased susceptibility to infection.

### 2.3 Vaccine formulations and protocol

Vaccines were formulated with antigens derived from Bucl8 that were predicted *in silico* to contain the extracellular loops 1 and 2 (L1 and L2) (22). Both synthetic loop-based peptides were conjugated separately to CRM_197_ (Cross-reacting material 197) by FinaBiosolutions, as described (22). L1-/L2-CRM_197_ (12.5 µg each in 100 µL) were combined with AddaVax (1:1 vol/vol in a 200-µL total volume) for the subcutaneous immunization/challenge study or L1-CRM_197_ (25 µg) with FCDG (fluorinated cyclic diguanosine monophosphate; 10 µg; InvivoGen, San Diego, CA, USA) in 40 µL for the intranasal immunization/challenge. Control groups included mice administered with the equivalent volumes of PBS or adjuvant only. Of note, *B. thailandensis* has a Bucl8-like protein similar to that of *B. pseudomallei* and *B. mallei* that contains identical surface-loop epitopes, L1 and L2, but does not have the collagen-like extracellular domain (24). Mice were immunized subcutaneously or intranasally three times, 21 days apart, with antigen-adjuvant formulations. Blood, spleens, and lungs were harvested 14 days after the final booster for select mice (-1 Days Post Infection; -1 DPI) to evaluate antibody types and titers, cell populations, and cytokine responses for immunogenicity studies. Remaining mice were challenged with bacteria.

### 2.4 Mouse infection protocol

Mice were challenged with either Bt E264 or Bt E264-*lux* at indicated doses and sample sizes in the figure legends and Results. The inoculum was prepared by diluting an overnight broth culture of Bt E264/-*lux* to an OD_600nm_ of 0.05 in LBM and grown to OD_600nm_ of 0.4 (~10^7^ CFU/mL). Cultures were centrifuged and washed once with cold PBS, then, pelleted; bacterial cells were suspended in saline to the desired inoculum size; bacteria plating was completed after inoculation. Infected mice were monitored for pre-/moribund symptoms and euthanized at humane endpoints or at 7 DPI endpoint. Humane endpoint criteria were based on a combination of a 20% drop in weight, body temperature below 33.5°C, severe decrease in activity, grooming, and well-being, trouble breathing, and/or high luminescent signal in the chest. Abdominal temperature was measured *via* an infrared thermometer.

### 2.5 Sample collection

Blood samples were obtained *via* cardiac puncture and processed in serum microtainer tubes as per manufacturers guidelines (BD). Lung supernatants were prepared by mechanically homogenizing lungs in 3 mL of complete medium, centrifuging at 1,000g for 7 mins, and aspirating the supernatant. Nasal wash samples were collected by pipetting directly into the nares and washing with ~120 µL of sterile PBS. Saliva was collected by pipetting sterile PBS between the cheeks and teeth, washing with ~120 µL. A protease inhibitor cocktail (Pierce, ref#A32955) was added per manufacturer’s instructions to the nasal wash and saliva. Bacterial burden in the nasal wash, blood (pre-centrifugation), spleen and lung tissues was assessed by serial 10-fold sample dilutions in PBS, plating in triplicate on Luria agar, and incubating at 37°C for 24 hrs.

### 2.6 Analysis of antibody responses by ELISA

Antigen-specific IgG, IgG subclasses, and IgA antibody responses were measured by ELISA. Wells were coated with 1 µg of unconjugated L1-peptide in 100 µL bicarbonate buffer for 2 hrs at room temperature, then blocked overnight at 4°C with 200 µL of 1% bovine serum albumin (BSA) in tris-buffered saline (TBS). Samples (sera, lung supernatant, nasal wash, saliva) were diluted 1:50 in 100 µL 1% BSA/TBS, added to wells, and incubated for 2 hrs at 37°C. For serum titers, antibody was serially diluted two-fold in 1% BSA/TBS. Antigen-bound antibody was detected with goat anti-mouse antibody alkaline-phosphatase-conjugate (Southern Biotech, 1:1000 dilution 1% BSA/TBS), incubated at room temperature for 1 hour, and alkaline-phosphate substrate (PNPP; Thermo Scientific) added. Seroconversion in mice immunized with antigen/adjuvant combination was compared to that recorded in control mice treated with adjuvant (AddaVax or FCDG).

### 2.7 Surface recognition of Bucl8 on *Burkholderia* cells

Whole-cell antibody binding was assessed by ELISA. Wells were coated with ~5 x 10^7^ cells, either with Bt E264-*lux* or Bp82, and incubated overnight at 4°C. Wells were washed with 0.05% Tween-20/PBS and blocked with 1% BSA in 0.05% Tween-20/PBS at 4°C overnight. Assay was completed as above.

### 2.8 IVIS *in vivo* imaging

Mice were imaged for bacterial luminescent signal using IVIS SpectrumCT *In Vivo* Imaging System (PerkinElmer). Mice were anesthetized with 3% isoflurane before and during imaging and imaged with medium binning for 3 minutes in supine position. Relative light units (RLU) for the chest, head, or whole body were calculated using Living Image Software and drawing boxes over the regions of interest. Background levels of luminescence were determined by drawing a region of interest on the imaging mat.

### 2.9 Flow cytometric analyses with lung homogenates

Specific cell populations in lung homogenates were analyzed by flow cytometry. An altered cell isolation protocol of (25) was used. Briefly, lungs were harvested and homogenized *via* mechanical force and strainer. Samples were dissociated in buffer (100 mg/mL collagenase D and 10 μg/mL DNase I in Hank’s buffered salt solution) for 45 minutes at 37°C, vortexing periodically.

Cells were stained using either a myeloid or lymphocyte cell panel described in **Table S1**. Approximately 10^6^ lung cells per well were seeded in a 96-well plate, centrifuged, and resuspended in 25 μL of Fc block (1:400 dilution of non-specific immunoglobulins in FACs buffer (5% fetal bovine serum in PBS)) for 15 minutes on ice. 25 μL of antibody were added to appropriate wells and stained for 20 minutes on ice, protected from light. Cells were washed, fixed with 1% paraformaldehyde, and assayed within a week. Samples were run on a BD LSRFortessa and analyzed using FCS Express 7 software.

All samples were gated to exclude high FSC/SSC, doublets (FSCA by FSCW), and then dead cells (Live/Dead stain+). For the lymphoid panel, populations were gated on CD11b- cells and then on respective marker populations (CD4+ vs CD8+, CD4+ vs B220+). For the myeloid panel, cells were gated on CD11b+ vs CD11c+ axes, isolating CD11b+ CD11c- cells and CD11b+ CD11c+ cells. Neutrophils and inflammatory monocytes were distinguished from CD11b+ CD11c- cell populations by Ly6C and Ly6G markers, indicated in **Table S2**. Macrophages and dendritic cells were gated from CD11b+ CD11c+ MHCII+ populations and distinguished by the presence of F4/80.

For lung cytokine analysis, each aliquot of lung homogenate, pre-dissociation, was centrifuged and supernatant collected and stored at -20°C. Protein cytokine levels were analyzed using a flow-cytometric bead assay per manufacturer’s instructions (Biolegend LEGENDplex™), which includes IL-2, IL-4, IL-5, IL-6, IL-9, IL-10, IL-13, IL-17A, IL-17F, IL-22, IFN-γ and TNF-α. Samples were run on a BD LSRFortessa and analyzed with LEGENDplex™ Data Analysis Software Suite.

### 2.10 Statistics

Statistical tests (Student’s *t*-test, One-way ANOVA, Two-way ANOVA, Spearman and Pearson correlations, and Principal Component Analysis, PCA) and *post-hoc* tests were performed using Graphpad Prism 9 software. Technical replicates for ELISAs and CFU quantification were completed in triplicate independent assays and flow cytometric assays in duplicate.

## 3 Results

### 3.1 Development of an outbred murine model of acute *Burkholderia* infection

**Figure 1:**
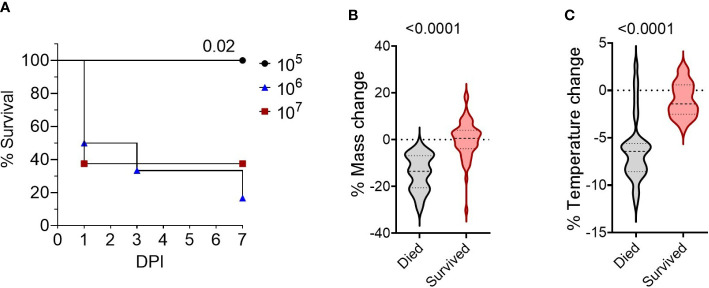
Survival of CD-1 mice intranasally infected with Bt E264. **(A)** Kaplan-Meier survival curves from representative dosing experiment. Groups (n= 6-8) of female and male mice were infected intranasally with increasing doses (2.9 x 10^5^ CFU (n=6, female/male n=3), 2.1 x 10^6^ CFU (n=6, female/male n=3), 2.5 x 10^7^ CFU (n=8, female/male n=4)) of Bt E264. Log-rank test. Mouse endpoint **(B)** weight and **(C)** temperature change during Bt E264 infection relative to starting value. Body weight and temperature of mice were recorded daily. Both parameters were graphed and compared statistically between mice that succumbed to disease vs those that survived from all experiments (n _died_ =34, n _survived_ =55, 4 experiments). Percent mass and temperature change were calculated by dividing a mouse’s end point measurement by the 0 DPI measurement. Student’s t-test. P values are shown.

To develop a BSL-2 respiratory *Burkholderia* infection model with outbred mice, we first established the infectious dose of *B. thailandensis* E264 (Bt E264) in CD-1 mice by testing three inocula of ~10^5^- ~10^6^ - ~10^7^ CFUs, based on infectious doses previously used for C57BL/6 and BALB/c inbred mice (19, 26). Mice that were infected with 10^5^ CFU survived for the duration of the study (**Figure 1A**) and cleared any visible symptoms by 7 days post-infection (DPI). Increasing the dose resulted in significant decreases in mouse survival rates within the 7-day experiment timeframe, indicating a more acute infection. We were interested in modeling the disease presentation during 1-7 DPI to assess the early correlates of protection. We observed that female mice were more affected by infection than males, with all females inoculated with 10^6^ and 10^7^ CFUs succumbing to infection by day 6 post-infection, although differences were not statistically significant. As expected, there was a significant difference in endpoint weight (**Figure 1B**) and temperature loss (**Figure 1C**; p < 0.0001, n_died_ =34, n_survived_ =55) between mice that succumbed to infection and those that survived (data compiled from four separate experiments, **Figure S1**). There were also significant differences in weight and temperature at the height of infection (3 DPI, **Figure S1**). Thus, these parameters were used to establish morbidity status in subsequent studies.

**Figure 2:**
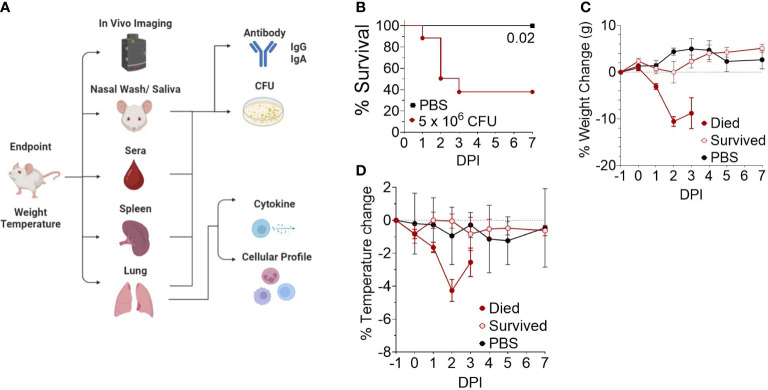
Development of an outbred BSL-2 acute model of respiratory *Burkholderia* infection. **(A)** General schematic of sample processing used to establish the correlates of protection in the model. Created with BioRender.com. **(B)** Kaplan-Meier survival curves. Female CD-1 mice were infected intranasally with an infectious dose (5 x 10^6^ CFU, n=21) of Bt E264-*lux* strain or mock-infected with PBS (n=4). Mice were euthanized at moribund or 7 DPI endpoints. **(C)** Weight and **(D)** temperature change over time, separated by survival group (n _1DPI_ = 3, n _2DPI_ = 9, n _3DPI_ = 3, n _7DPI_ = 6, n _PBS_ = 4).

We next investigated the disease time-course utilizing *in vivo* IVIS imaging to track the spread and intensity of infection along with other model parameters illustrated in **Figure 2A**. Female CD-1 mice were intranasally inoculated with 5 x 10^6^ CFU Bt E264-*lux* strain, resulting in 44% survival (**Figure 2B**), while control mice were administered PBS. Decreases in weight and temperature peaked at 2-3 DPI (**Figures 2C, D**), which corresponds to the decrease in survival (**Figure 2B**). Mice were separated into groups based on survival to underscore that surviving mice had low variation in temperature and weight throughout the course of infection. PBS mice had a slight increase in weight, which leveled at 5-7 DPI (**Figure 2C**).

**Figure 3:**
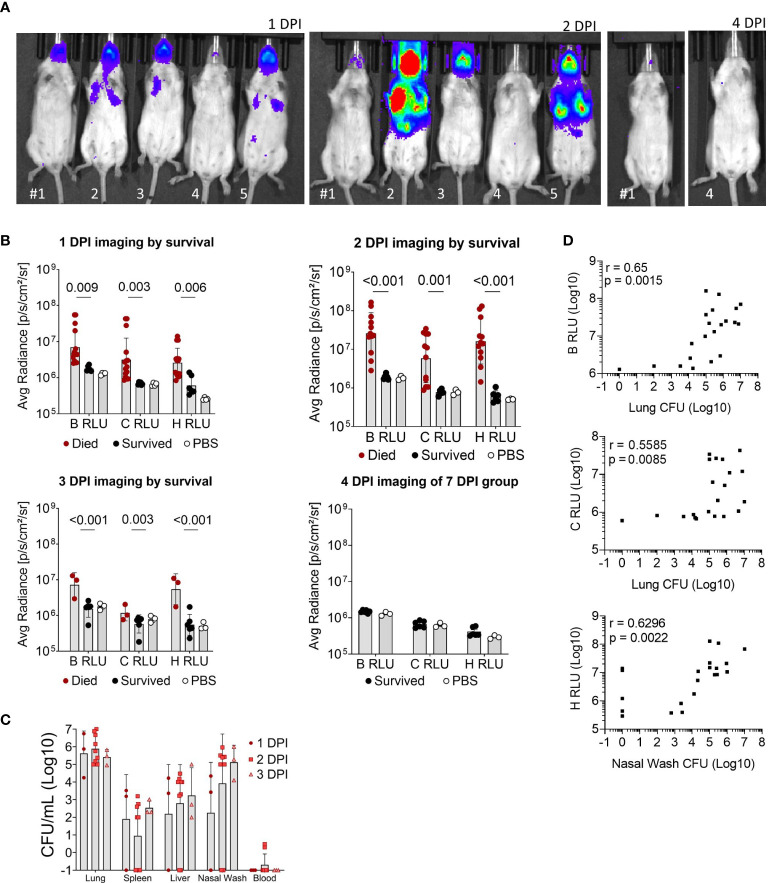
Luminescent imaging of CD-1 mice infected with BtE264-*lux*. **(A)** Depiction of *in vivo* IVIS imaging. Representative mice (labeled #1-5) are shown that were imaged at 1 DPI, 2 DPI, and 4 DPI. Mice #2, 3, and 5 were euthanized after 2 DPI imaging due to moribund symptoms. **(B)** Quantification of *in vivo* bioluminescence. Luminescent signals were measured for whole body (B RLU), chest (C RLU), and head (H RLU). Two-way ANOVA with Šídák’s multiple comparison test on log-transformed data. **(C)** Bacterial burden in blood and organs. Lung homogenates, spleen, liver, nasal wash, and blood were plated to assess bacterial presence (CFU) in the specimens. **(D)** Correlations between bacterial burden and bioluminescence. Spearman correlations between CFUs of lung homogenate or nasal wash with luminescent signal are shown. Geometric means and standard deviation are shown for **(B, C)**. Images and samples are from animals from **Figure 2**; n _1DPI_ = 3, n _2DPI_ = 9, n _3DPI_ = 3, n _7DPI_ = 6, n _PBS_ = 4.

Luminescent signal from bacteria was also measured for the whole body (B RLU), or the chest (C RLU) and head (H RLU) regions separately on 1, 2, 3, 4 and 7 DPI (**Figures 3A, B** **)**. Representative images of the luminescent signal progression for mouse head and chest regions are shown in **Figure 3A** and corresponding data in **Figure S2**. Mice were separated by their survival group and luminescence quantified for each region in order to highlight the heterogeneity of the primary infection (**Figure 3B**); those mice that initially had a high luminescent signal became moribund, while those with low luminescent signal survived. The mice that survived were not significantly different from mock-infected mice throughout the course of infection (**Figure 3B**). Of note, few mice had concentrated signal in the lower abdomen (**Figure 3A**, panel 2 DPI, mice #2 and #5), which likely indicated bacterial spread to other organs; however, we did not sample these areas.

Nasal wash, blood, liver, spleen, and lungs were examined for bacterial presence (**Figure 3C**); all non-surviving mice had bacteria in the lungs and varying bacterial burden in other tissue specimens. Surviving mice did not have measurable CFUs in any tissue and were not graphed. Several moribund mice at 3 DPI had detectable bacteria in the spleen, liver and blood, indicating systemic spread. Luminescent signal for all mice began to increase at a bacterial burden of ~5 x10^3^ CFU or greater in the nares and ~10^5^ CFU in the chest (**Figure 3D**). The bacterial burden (CFUs) in lungs significantly correlated with the B RLU (0.65, p < 0.001) and C RLU (0.56, p < 0.01) intensities, and in the nasal wash with the H RLU (0.63, p < 0.01).

**Figure 4:**
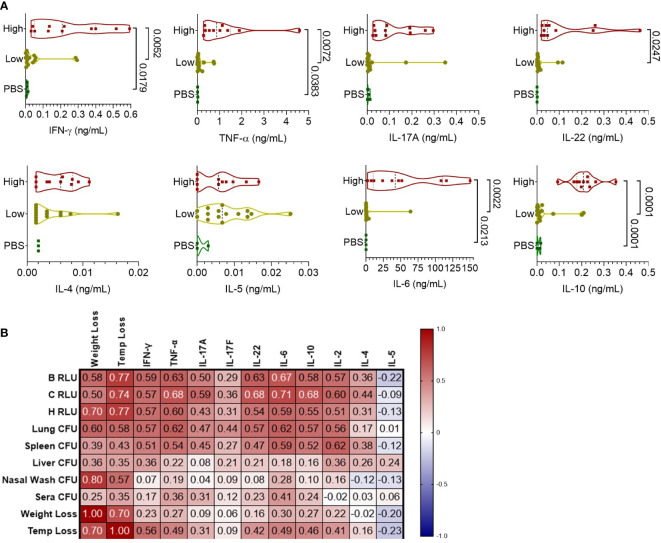
Pulmonary cytokine response of CD-1 mice infected with BtE264-*lux*. **(A)** Cytokine levels during infection. Lung homogenate supernatants were analyzed for protein levels of Th1/Th17/Th2 cytokines using a bead-based flow cytometry assay. Mice were separated into either a high-burden group based on moribund status and/or presence of luminescent signal in the lungs, or a low-burden group if moribund symptoms were absent or luminescent signal was only detected in the nares. One-way ANOVA with Tukey’s multiple comparison test. P values are shown. **(B)** Spearman correlations between cytokines and disease indicators. n _1DPI_ = 3, n _2DPI_ = 9, n _3DPI_ = 3, n _7DPI_ = 6, n _PBS_ = 4.

To understand the cellular response to infection, cytokine levels from the supernatant of lung homogenates were measured and categorized based on high bacterial burden (luminescent signal in the lungs), low-burden (only nasal or no luminescent signal), or control PBS mice (**Figure 4A**). Mice with a high luminescent signal expectedly had significantly increased levels of inflammatory cytokines IFN-γ, TNF-α, IL-22, and IL-6 compared to low burden and PBS mice. TH2-based cytokines IL-4 and IL-5 were increased at similar levels for the high and low burden groups. Notably, measurements of the anti-inflammatory cytokine IL-10 was significantly increased in the high-burden group. In context of disease indicators of all mice, Th1 and Th17 cytokines were positively correlated with disease burden (**Figure 4B**), especially with luminescent burden and lung/spleen bacterial burden. Luminescent signal from the chest correlated strongly with tissue-damaging cytokines (IL-6, TNF-α, IL-22) and IL-10 (0.68-0.71, p < 0.0001).

Altogether, here, we developed an outbred CD-1-mouse model of early respiratory *Burkholderia* infection, using the BSL-2 *B. thailandensis* strain E264-*lux* as a surrogate organism for *B. pseudomallei*, which we used in this study to identify and assess the early correlates of vaccine-elicited protection.

### 3.2 Subcutaneous immunization with Bucl8-based vaccine does not protect from respiratory challenge

Our previous studies have shown that both CD-1 and C57BL/6 mice elicited robust TH2-antibody responses following subcutaneous vaccination with Bucl8-based synthetic loop peptides L1 and L2 (vaccine schedule shown in **Figure S3** **(** 22 **),**). Groups of 4-5-week-old female and male CD-1 mice were subcutaneously immunized thrice with vaccine formulation containing 1:1 mixed L1 and L2 peptides - each conjugated with CRM_197_ - and adjuvanted with AddaVax (n=14) or AddaVax-only controls (n=12). We reported that this vaccine formulation elicited high levels of IgG1 antibodies and a moderate level of IgG2a/b, indicating a TH2-biased immune response in both CD-1 and C57BL/6 mice (22). Here, we show that when CD-1 immunized mice were intranasally challenged with Bt E264 strain (8 x 10^6^ CFU), the L1/L2-based vaccination did not increase protection from death compared to AddaVax-only controls (**Figure S3B**), nor produced significant differences in weight or temperature changes (data not shown).

### 3.3 Intranasal immunization with Bucl8-based vaccine ameliorates disease indicators in CD-1 mice after intranasal instillation of *B. thailandensis*

Subcutaneous vaccination did not provide protection in spite of the high titers of antigen-specific antibodies, therefore we changed our approach to better prime the respiratory tract and promote a more Th1-based immune response. Thus, we altered the route of immunization to intranasal and the adjuvant to a mucosal adjuvant (fluorinated cyclic diguanosine monophosphate; FCDG) that stimulates the STING (stimulator of interferon genes) pathway and drives cytotoxic immunity (27–30). We used the same vaccination schedule as before (**Figure S3**), where mice were intranasally immunized thrice, three weeks apart, with L1-CRM_197_ plus FCDG or with FCDG only; we used the L1-only formulation based on higher antibody titers and higher predictive immunogenicity than L2, as shown in our previous data (24). Following the last immunization, some mice were euthanized prior to infection (-1 DPI, n=5 per treatment) to examine baseline levels of antigen-specific antibodies, lung cytokines, and cell populations, while remaining mice (n _1-3 DPI_ =6, n_7 DPI_ =7 per treatment) were intranasally challenged with Bt E264-*lux* (8 x 10^5^ CFU).

**Figure 5:**
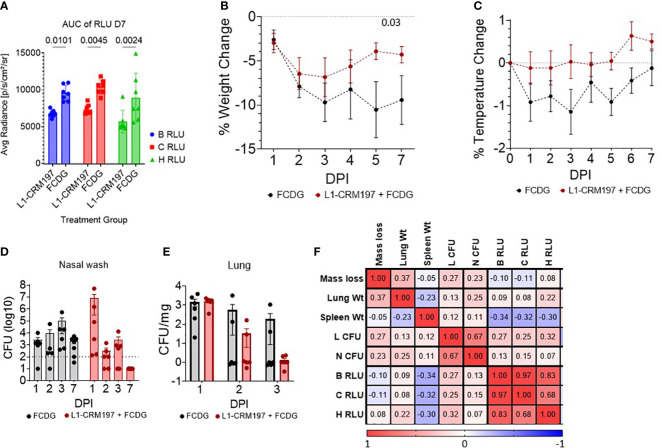
Disease parameters in intranasally vaccinated mice challenged with Bt E264-*lux*. 4-5 wk old female CD-1 mice (n _-1 DPI_ =5, n _1-3 DPI_ =6, n _7 DPI_ =7 per treatment) were immunized with either L1-CRM_197_ + FCDG or FCDG-only three times and then challenged with 8 x 10^5^ CFU Bt E264-*lux*. **(A)** Luminescent signal of Bt E264-*lux* loads. IVIS measurements were taken for the whole mouse body (B RLU), chest (C RLU) and head (H RLU) areas on 1-4, and 7 DPI. Area under the curve (AUC) of RLU for all days is shown for 7 DPI mouse group. Student’s *t*-test. P values are shown. Percent **(B)** weight and **(C)** temperature change over time of all mice immunized with L1-CRM_197_ + FCDG (red line) or FCDG-only control (black line). Dotted line at zero represents starting weight. P value shown; RM-Two way ANOVA. Bacterial burden in **(D)** nasal washes and **(E)** lung homogenates. Dotted line represents limit of detection. Lung CFUs were normalized to lung tissue weight. **(F)** Analysis of disease parameters. Spearman correlations were assessed, with positive correlations in red and negative correlations in blue. CFU; colony forming units. RLU; relative light units. Wt; weight. Day post infection; DPI. Geometric means and standard deviation are shown for **(D, E)**.

There were significant differences between treatment groups when comparing the area under the curve (AUC) of luminescent signals over time of mice that survived the whole experiment (7 DPI groups, **Figure 5A**). Luminescent signals were not significantly different between treatment groups until D4 of imaging (**Figure S5**). Additionally, luminescent levels of all regions of interest (B RLU, C RLU, and H RLU) correlated moderately to strongly with each other (>0.68, **Figure 5F**), indicating infection in the nasal area corresponded with lung infection.

There was a significant change in weight over time for both experimental groups (p < 0.001, RM Two-way ANOVA). All mice initially lost weight during days 1-3 post-infection, then, mice vaccinated with L1-CRM_197_ antigen regained weight by day 7, whereas mice in the control FCDG-only group did not (**Figure 5B**). FCDG-only mice had a non-significant decrease in temperature as well (**Figure 5C**).

Endpoint bacterial loads in nasal washes for the L1-CRM_197_ group significantly decreased by 6 logs during days 1-7 post-challenge (p < 0.001, log_10_ transformed, Two-way ANOVA) and were significantly different from FCDG-only group 7 DPI (p < 0.017, **Figure 5D**). We note that three mice in the L1-CRM_197_ group had higher bacterial burdens than the FCDG-only control at 1 DPI, while the other three mice did not. Bacteria were cleared in lungs of L1-CRM_197_ immunized mice, but not in the FCDG-only control group by 7 DPI (**Figure 5E**). Additionally, nasal and lung bacterial burdens positively correlated with each other (0.67, **Figure 5F**), further supporting that high-burden mice were affected in both areas. Lung and spleen weights were also recorded and differences between treatment groups were not significant (**Figure S4**); however, spleen weight negatively correlated with the luminescent burdens (**Figure 5F**), suggesting mice, regardless of treatment, that had increased spleen weight had decreased burdens.

### 3.4 Intranasal immunization with a peptide-conjugate elicits mucosal and systemic antigen-specific antibodies

**Figure 6:**
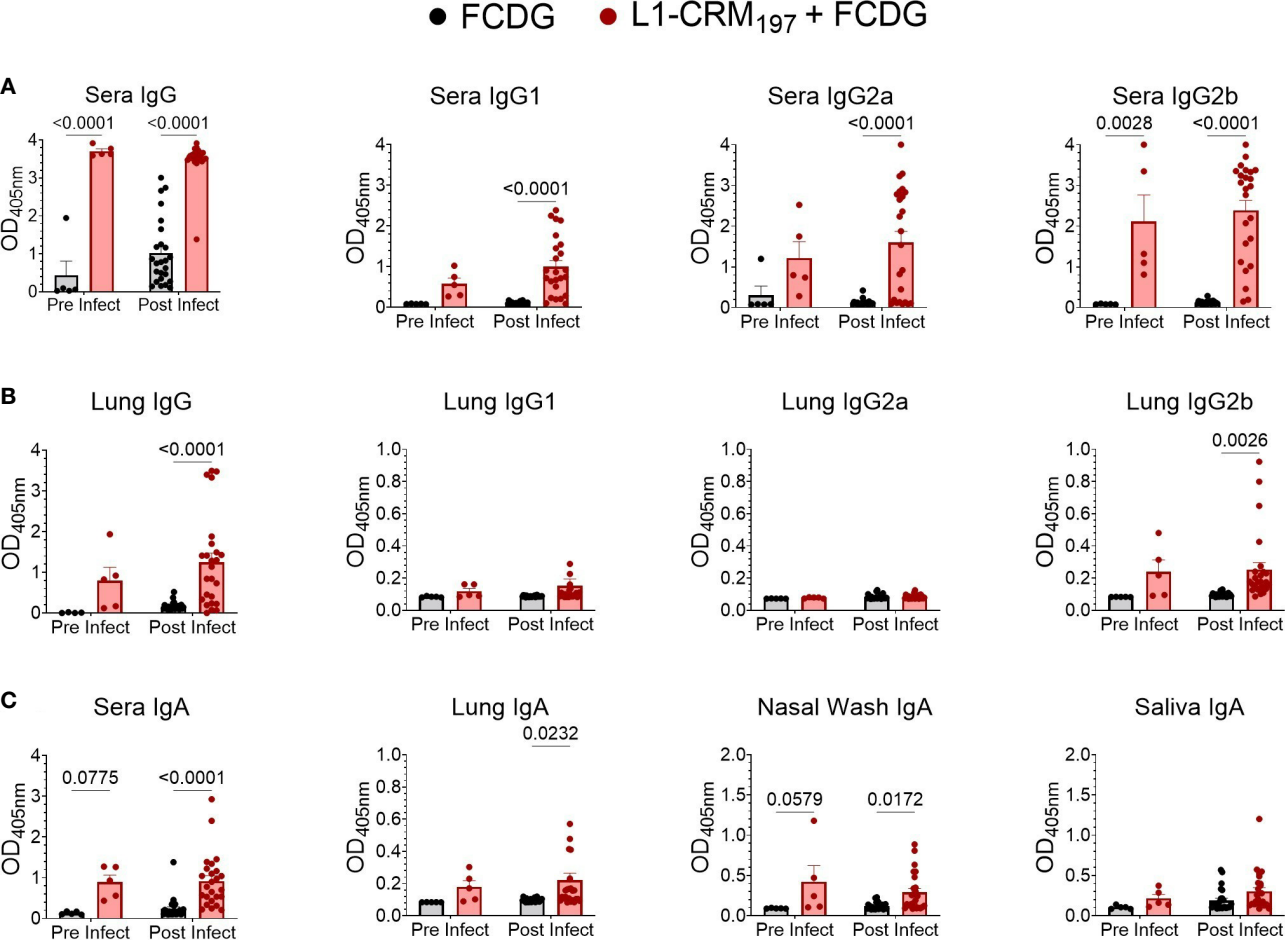
Intranasal vaccination with L1-CRM_197_ antigen elicits specific humoral and mucosal immune responses. Specific antibodies again L1 antigen were measured by ELISA following intranasal vaccination (pre-infect) and after Bt E264-*lux* challenge (post infect). Samples are from animals from **Figure 5**. Wells were coated with unconjugated L1-peptide to detect the antigen-specific Ab, as follows: **(A)** total IgG and IgG subtypes in sera), **(B)** IgG and IgG subtypes in lung supernatants, **(C)** IgA in sera, lung supernatants, nasal washes, and saliva. Two-way ANOVA with Šídák’s multiple comparison test. P values are shown. n _-1 DPI_ =5, n _1-3 DPI_ =6, n _7 DPI_ =7 per treatment.

Although high anti-L1 antibody titers did not result in protection when mice were subcutaneously immunized [(22), **Figure S3B**], changes in immunization route and adjuvant type can alter whether protective antibodies are produced. Following intranasal vaccination with L1-CRM_197_ plus FCDG, we assayed mouse sera and lung homogenates for L1-specific total IgG, IgG1, IgG2a, and IgG2b subtypes (**Figures 6A, B**). In addition, mucosal immunity was assessed by detecting L1-specific IgA in sera, lung homogenates, nasal washes, and saliva (**Figure 6C**). Vaccination with L1-CRM_197_ induced seroconversion to Bucl8-L1 antigen, indicated by high levels of total IgG and IgG subtypes detected in wells coated with L1-peptide *via* ELISA when comparing pre (-1 DPI) and post (1-7 DPI) infection sera (1:50 dilution) (**Figure 6A**). Antibody levels were further assessed *via* titrating sera two-fold (**Figures S6A, B**). In general, sera from mice vaccinated with L1-CRM_197_ continued to have positive immunoreactive signal through 1:3200 dilution. Interestingly, mice from the L1-CRM_197_ 1 DPI group had significantly reduced titers compared to the prior or following days (**Figure S6B**); these sera largely originated from sacrificed moribund mice, which could indicate that mice that did not elicit high L1-specific antibody levels were not protected from progressing infection. In contrast, the immunoreactive signal detected in FCDG-control sera dropped at 1:100 dilution and continued to decline to background levels until 1:200-1:800 dilution. Additionally, although naïve to L1-peptide, FCDG mice had increasing titers to L1-peptide as the days progressed.

Virtually all L1-vaccinated mice had IgG2b against L1-peptide, but the IgG2a and IgG1 responses were more variable. FCDG-control mice had minimal levels of total IgG against L1-peptide in pre-infection sera, but detection of L1-binding antibodies increased in post-infection samples, suggesting bacterial-stimulated seroconversion to Bucl8-derived antigen. (**Figure S6A**). There were significant differences between treatment groups for lung total IgG and specifically IgG2b in post-infection samples (**Figure 6B**), but not for IgG2a and IgG1 subclasses. This could suggest the antibody responses being produced locally in lung tissue are IgG2b. Additionally, vaccinated mice developed L1-specific IgA for all sample types (**Figure 6C**). IgA levels in sera and nasal washes were significantly different between treatment groups at both pre-and post-infection time points, while lung IgA levels were only significantly different post-infection. Mice had also detectable L1-specific IgA in saliva, but levels were not different between treatments.

Ratios of antibody types measured in sera and lung homogenates were compared to understand whether the antibody response was skewed towards one immune response and if infection altered these ratios (**Figures S6C, D**). When comparing Th2-associated IgG1 levels in mouse sera to either Th1-associated IgG2a or IgG2b in L1-vaccinated mice, the response was predominately skewed towards IgG2a/b (**Figure S6C**), while a similar effect was not observed between FCDG control groups. The ratio between IgG2a and IgG2b was mostly skewed towards IgG2b for serum and lung antibodies (**Figure S6D**).

**Figure 7:**
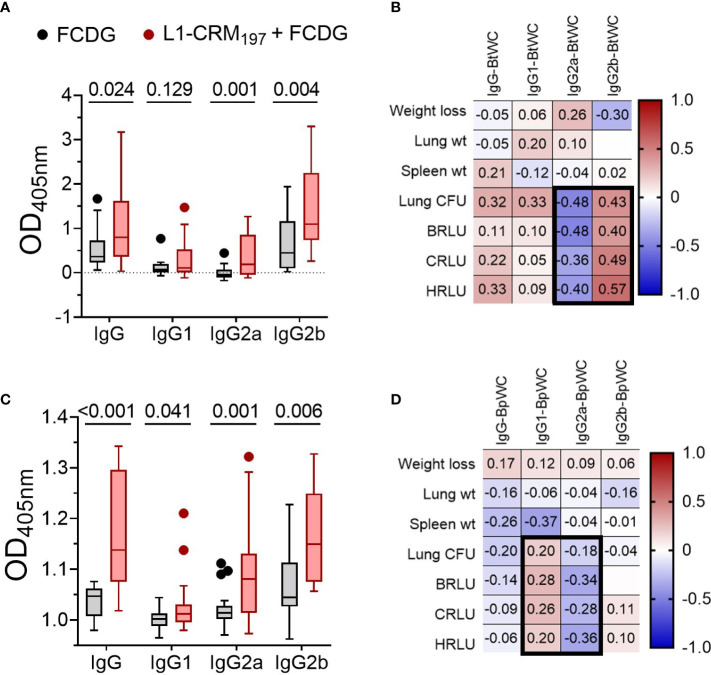
Antibodies from L1-vaccinated mouse sera recognize whole-cell bacteria. Binding *via* ELISA of sera IgG and IgG subtypes to wells coated with 10^7^ CFUs of **(A)** Bt E264-*lux* or **(B)** Bp82. Samples are from animals from **Figure 5**. Tukey boxplot with outliers shown. Student’s t-test. P values are shown. Pearson correlations between disease indicators (**Figure 6**) and whole cell antibody-binding levels for **(C)** BtE264 whole cell binding (BtWC) and **(D)** Bp82 whole cell binding (BpWC). n _-1 DPI_ =5, n _1-3 DPI_ =6, n _7 DPI_ =7 per treatment.

We next assessed by ELISA whether elicited antibodies would recognize the conserved Bucl8-L1 epitopes on whole bacterial cells of the surrogate *B. thailandensis* strain Bt E264-*lux* (**Figure 7A**) and *B. pseudomallei* Bp82, a BSL-2 derivative of the clinical isolate Bp 1026b (**Figure 7B**). Compared to FCDG controls, the vaccinated mice had significant increases in total IgG, IgG2a, and IgG2b against whole-cell Bt E264 and minimal of IgG1 (**Figure 7A**). Similarly, L1-immune sera recognized whole Bp82 cells, which were principally IgG2a and/or IgG2b (**Figure 7B**). Overall, intranasal vaccination with L1-CRM_197_ elicited specific antibodies against L1-peptide, both mucosal IgA and systemic IgG that were predominantly IgG2b. Importantly, elicited antibodies recognized Bucl8-L1 antigen on bacteria cells indicating surface exposure of the loop 1 sequence postulated based on the Bucl8 model (22). Although not vaccinated with L1-CRM_197_, some mice of the control FCDG-only group seroconverted to L1 (post-infection samples), thus, suggesting possible seroconversion due to the host’s exposure to Bucl8-L1 antigen expressed *in vivo* during Bt E264 infection – these antibodies also recognized Bucl8-L1 epitope on Bp82, which was envisaged conceptually. Furthermore, low bacterial burdens in lungs, as assessed using IVIS and by plating, correlated with higher IgG2a binding to Bt E264 (p < 0.05; **Figure 7C**). In contrast, mice that had high bacterial burdens positively correlated with IgG2b binding (p < 0.05). Correlations of Bp82-binding with bacterial burdens were weaker than that of Bt E264, however IgG2a still had a negative correlation (**Figure 7D**), suggesting the antigen-specific IgG2a elicited could be protective against Bp82 as well.

### 3.5 Vaccination with L1-CRM_197_ dampens the inflammatory cytokine response

**Figure 8:**
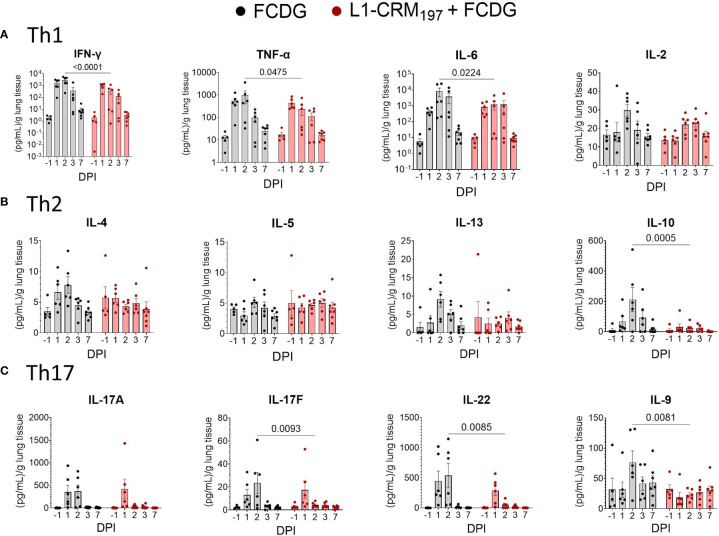
Intranasal infection elicits a Th1/17 dominant immune response in the lung environment. Cytokine protein levels were measured in lung homogenate supernatants from mice immunized with L1-CRM_197_ + FCDG (red) or FCDG (black) and challenged with Bt E264-*lux*. Samples are from animals from **Figure 5**. **(A)** Th1 inflammatory cytokine levels (IFN-γ, TNF-α, IL-6, IL-2). **(B)** Th2 B-cell memory and anti-inflammatory cytokine levels (IL_4, IL-5, IL-13, IL-10). **(C)** Th17 inflammatory and regulatory cytokine levels (IL-17A, IL17F, IL-22, IL-9). One-way ANOVA with Šídák’s multiple comparison test. P values are shown. Day post infection; DPI. n _-1 DPI_ =5, n _1-3 DPI_ =6, n _7 DPI_ =7 per treatment.

To evaluate the cellular response to infection, we measured T helper cell cytokines from the lung homogenate supernatants. Both treatment groups had a Th1-type inflammatory response to infection, represented by IFN-γ, TNF-α, and IL-6 cytokines, that peaked on 2 DPI and returned to pre-infection levels by 7 DPI (**Figure 8A**). The FCDG-control group had significantly increased levels of all 3 cytokines on 2 DPI compared to the L1-vaccinated group. IL-2 is a T cell proliferation cytokine (31) that was not significantly different between groups, but similarly increased at the height of infection. Th2 cytokines IL-4, IL-13, and IL-10 also peaked on 2 DPI in the FCDG-only control group, but not in the L1-CRM_197_-vaccinated group (**Figure 8B**). Of note, the anti-inflammatory IL-10 cytokine was minimally produced by the L1-CRM_197_-group over the time of experiment; IL-10 levels were significantly different at the peak of inflammatory response on 2 DPI compared to the FCDG-only mice. Th17 cytokines IL-17A/F and IL-22 sharply peaked during 1-2 DPI in the control group, and on 1 DPI in the L1-CRM_197_ -vaccinated group (**Figure 8C**); the levels of these cytokines were significantly lower in L1-CRM_197_ -vaccinated mice on 2 DPI compared to FCDG-control mice, including IL-9, which showed somewhat different course of expression. Overall, L1-CRM_197_ -vaccinated mice had decreased inflammatory cytokine responses to bacterial infection, especially on 2 DPI.

All the inflammatory cytokines strongly correlated with each other (0.71 to 0.94, p < 0.001) and with IL-10 and IL-4 cytokines (0.54 to 0.77, p < 0.001) (**Figure S7**). Bacterial luminescence and bacterial counts in the lungs also correlated with inflammatory cytokines (0.34 to 0.70, p < 0.05). Of the lung cytokines measured, only IL-9, IL-13, and IL-2 (p < 0.05) had negative correlations to disease indicators, in particular with the luminescent burdens (**Figure S7**). Additionally, IL-13 correlated with IL-9 (0.43, p < 0.01) and IL-2 (0.64, p <0.001) when evaluating all mice together. When correlations were separated based on vaccination status (**Figures S8**; **9**), IL-9 was the only cytokine negatively correlated for both treatments of these three cytokines (-0.24 to -0.36 RLUs L1-CRM_197_, p < 0.05; -0.35 to -0.38 RLUs FCDG, p < 0.05).

### 3.6 *B. thailandensis* challenge increases inflammatory cells in the lungs

**Figure 9:**
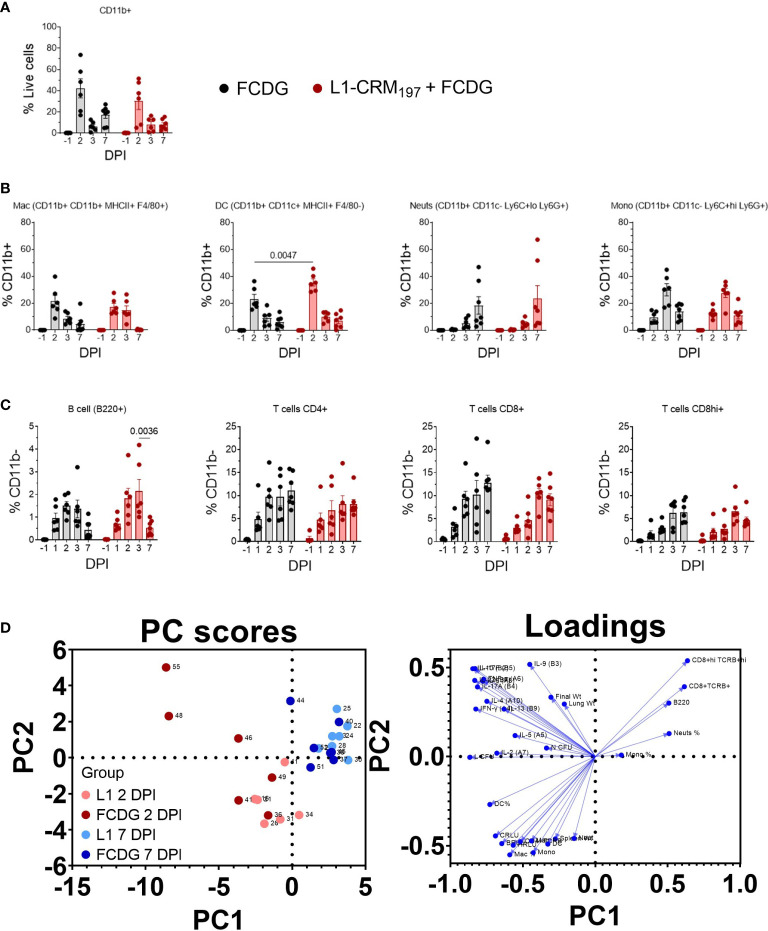
Infiltrating myeloid and lymphoid populations increase with inflammation. Myeloid and lymphoid cell populations were measured from lung homogenates *via* flow cytometry. Samples are from animals from **Figure 5**. **(A)** Percentage of myeloid (CD11b+) cells from total cell population. **(B)** Percentage of macrophages (Mac), dendritic cells (DC), neutrophils (Neuts), and monocytes (Mono) from CD11b+ population. Cell labels are indicated. **(C)** Percentage of B cells (B220+), T cells (CD4+, CD8+lo, or CD8+hi) gated on CD11b- cells. P values shown. Two-way ANOVA with Šídák’s multiple comparison test. **(D)** Principal components analysis (PCA) scatterplot. Points represent individual mice. Levels of cytokines, cell populations, and disease indicators are colored by 2 (red) and 7 DPI (blue) groups and separated by vaccination status (light vs dark colors). Loadings of variable inputs are shown. Day post infection; DPI. n _-1 DPI_ =5, n _1-3 DPI_ =6, n _7 DPI_ =7 per treatment.

To further characterize the cellular response to infection, we measured changes in myeloid and lymphoid cell populations in lung homogenates *via* flow cytometry. Cells were first gated on CD11b+ cells and then differentiated by cell markers. All cell populations were at minimal or non-detectable levels pre-infection. Post-infection, the CD11b+ population significantly increased 2 DPI for both treatment groups (p < 0.01, **Figure 9A**), and then decreased 3 and 7 DPI to a level near baseline (-1 DPI). The majority of myeloid cells during early infection for both treatment groups were macrophage (12-27% L1-CRM_197_, 8-39% FCDG-only) and dendritic cell (28-45% L1-CRM_197_, 15-36% FCDG-only) populations (**Figure 9B**). The L1-peptide vaccinated group had a significantly increased population of dendritic cells 2 DPI compared to the FCDG-only group (p < 0.0047). Higher dendritic cell ratios correlated positively with increased levels of cytokines (0.14-0.5, p < 0.05), chest luminescent signal (0.47, p < 0.01), and lung bacterial burden (0.71, p < 0.001), indicating dendritic cells may be secreting more inflammatory cytokines and/or increase with bacterial burden (**Figure S10**). Macrophages also correlated positively with chest luminescent signal (0.54, p < 0.001), IFN-γ (0.36, p < 0.03), and Th2/mucosa-based cytokines IL-2, IL-4, and IL-13 (0.33-0.40, p < 0.05). Thus, dendritic cells are associated with inflammation and macrophages with Th2/mucosal cytokines. In comparison to macrophages and dendritic cells, neutrophils were a smaller percentage of the early myeloid cell population (< 11%), but increased for both treatment groups 7 DPI. Monocyte populations were approximately 10-15% of CD11b+ cells on 2 and 7 DPI, but significantly increased 3 DPI.

Lymphoid cell populations increased upon infection for both treatment groups, peaking at 2-3 DPI (**Figure 9C**). B cells were less than 5% of CD11b- cells, whereas CD4+ and CD8+ cells were ~10-20%. For the L1-vaccinated group, CD4+ T cells remained at similar levels post 2 DPI, while CD8+/hi cells increased slightly, indicating sustained T cell populations during infection resolution. Lymphoid populations correlated negatively with bacterial burdens (**Figure S10**), particularly chest burden (-0.25 to -0.58), with the CD8+hi population having the strongest correlation (-0.58 C RLU, p < 0.001; -0.41 lung CFU, p < 0.01). CD4+ cells positively correlated with Th2-type cytokines IL-5, IL-4, and IL-13 (0.30-0.40, p < 0.02-0.07), bacterial burden, and strongly with H RLU (0.63, p < 0.001). These data suggest that either lymphoid cell populations begin to increase a few days after infection and/or those mice that had lower burdens had increased lymphoid cells.

To garner an understanding of how individual mouse cell-mediated immune profiles related to others, we performed a principal component analysis (PCA) that included cell populations, cytokine levels, and disease indicators for individual mice (**Figure 9D**). We predominantly analyzed data from day 2 post infection, when most changes occurred, and data collected at the end of the experiment. PC1 contributed to 34.53% proportion of variance and PC2 to 14.31%. Mice mostly clustered by DPI group, where the 2 DPI groups (light and dark red points) had higher variability between and within treatment groups; FCDG-only mice (dark red points) were mainly separated from L1-vaccinated mice due to increased inflammatory cytokine levels. 7 DPI mice (blue dots) were clustered separately from the other groups, correlating with higher levels of neutrophil, monocyte, CD8+, CD8+hi, and B cell populations. This clustering is also driven by the lack of inflammatory cytokine levels. Overall, these data indicate that the status of infection (day 2 vs day 7) separated out as different clusters, with some vaccination effect seen at the peak of infection on day 2.

## 4 Discussion

Vaccine development against *B. pseudomallei* has been hindered by several factors, which include pathogen-specific obstacles, logistical difficulties, and regulatory barriers. *B. pseudomallei* are difficult to target because they are intracellular bacteria where antibody-based protection is variable, they are innately plastic making it difficult to target specific immunogens, and they have varying levels of virulence based on the route of infection. For example, aerosolized routes of transmission tend to be more serious compared to percutaneous routes (32), leading to pneumonia and rare cases of brain infection (33, 34). Further exacerbating the issue, *B. pseudomallei* infections can be asymptomatic, acute, chronic, or latent (33), with each type of infection most likely needing a different vaccine approach. For research purposes, two main murine models are used: BALB/c, which represent acute disease, and C57BL/6, which represent a chronic model. The purpose of these experiments was to (i) develop a BSL-2 level murine model to evaluate efficacy of a melioidosis vaccine proposed here and for others, and (ii) evaluate the efficacy of a Bucl8-based subcutaneous or intranasal vaccine.

### 4.1 Comparison of outbred model to existing inbred models

Prior melioidosis vaccine studies have used BALB/c or C57BL/6 models, which is well summarized by Wang et al. (3). BALB/c mice are considered an acute model because they are more prone to infection, while C57BL/6 mice are more resistant to initial disease and can succumb to infection from a few days post-infection to 60 days depending upon the strain and dose of bacteria and route of infection. Inbred mice should in theory elicit similar immune responses as they have the same genetic background. On the other hand, CD-1 mice are outbred and have been used in vaccines studies of other pathogens due to their genetic diversity (35, 36), providing a model to assess therapeutics against varying types of immunity.

Here, CD-1 mice responded in an acute manner, whereas intranasal infection with *B. thailandensis* resulted in severe infection of ~40% of mice within the first few days. Weight and temperature change showed that mice had a divided response from the onset of infection, either becoming pre- or moribund early on (2-3 DPI) or remaining comparatively unaffected during duration of experiment. Mice displaying limited morbidity in response to infection exhibited slight weight and temperature loss, as well as minimal bacterial burden as indicated by luminescent imaging and colony counts. As expected, mice with high bacterial burdens had greater weight and temperature loss, significantly increased bacterial burden and levels of inflammatory cytokines, as well as uniformly had increased IL-10 levels. As described, this model reflects the high variability in response to infection, which is also observed in melioidosis infections. Here, we propose the CD-1 *B. thailandensis* model as a prospective acute surrogate model of melioidosis to evaluate the efficacy of treatments that are dependent on variable host genetics, such as vaccine responses. Additionally, we note we did not examine timeframes past seven days, and thus these data are representative of the primary, acute pathology. Future directions can examine whether infection re-emergence occurs in CD-1 mice, and if this holds true for both *B. pseudomallei* and *B. mallei* infections.

### 4.2 Comparison of subcutaneous and intranasal vaccine antibody responses to a Bucl8-based antigen

Our prior *in vitro* studies have shown that transcription of genes in the *bucl8* locus are upregulated together in response to fusaric acid mycotoxin substrate in *B. pseudomallei in vitro* (21), and that sera from mice immunized with L1 peptide recognize whole cell *B. pseudomallei* (22). We also reported that putative homologous Bucl8-associated efflux pumps are present in *B. thailandensis* and the BCC organisms (24). Interestingly, sera from control mice immunized with FCDG and infected with Bt E264-*lux* elicited antibodies recognizing unconjugated L1-peptide, with increasing titers by 7 DPI, indicating the Bucl8-L1-epitope is being expressed in the absence of fusaric acid *in vivo* which is recognized by the immune system. Altogether, our data indicate immunized and naïve mice develop an immune response to the Bucl8-L1-antigen upon vaccination and infection, accordingly.

Additionally, we observed that the pre-moribund mice sacrificed at 1 DPI had lower levels of L1-specific IgG in the L1-vaccinated group suggesting that these mice - lacking high L1-specific antibody titers - were not protected at the start of infection, while sera from moribund but surviving mice collected during 2-7 DPI had significantly higher titers. This data set may support the hypothesis that antibodies are effective when infecting bacteria are extracellular, but become less effective when they are intracellular. We did not demonstrate when the bacteria switched from an extracellular to intracellular lifestyle, and thus further studies would be needed to determine the time course of the lifestyle transition.

While not studied extensively in *Burkholderia* infections, it has been noted that murine IgG2a may have protective properties, which is also supported in our data. For example, a subunit vaccine formulation based on the outer membrane protein OmpW elicited a higher IgG2a/IgG1 ratio, providing increased protection when paired with an oil-in-water emulsion adjuvant, SAS (9). In comparison, the same protein paired with the adjuvant alum elicited a higher IgG1/IgG2a ratio, with no detectable IgG2b, which had significantly decreased protection. Intranasal immunization with L1-CRM_197_ + FCDG led to significantly increased whole-cell-binding IgG2a antibodies in some mice, which correlated with decreased bacterial burden in those animals. IgG2a is cited as having complement-fixing and strong FcγR-mediated activity (37, 38); therefore, studies examining IgG2a in the context of FcR activation on neutrophils and macrophages, which are critical for both bacterial clearance and bacterial harboring of *Burkholderia*, may provide insight on the link between Th1/Th2 balanced, protective immunity and immune-tolerant effects. IgG2a is also a T-dependent antibody (37), making subunit immunogens ideal for driving the response. Thus, future vaccine formulations should consider targeting IgG2a production, which in our studies was promoted by the combination of the L1-based antigen, FCDG, and intranasal route.

Murine IgG1 has higher antigen affinity than IgG2a/b and binds with a higher affinity to FcγRIIb, which is an inhibitory Fc receptor on innate immune cells and leukocytes, specifically the only Fc receptor on B cells, and has anti-inflammatory activity (37). We previously compared antigen-specific IgG titers elicited from subcutaneous immunization with L1-CRM_197_ + AddaVax in CD-1 and C57BL/6 mice. Both murine models had an IgG1-dominant response against L1-peptide, complemented with a lesser IgG2a/b response (22). However, subcutaneous vaccination did not protect from death, following respiratory challenge with Bt E264 (CD-1 mice), nor did it significantly affect disease indicators. With intranasal vaccination IgG1 was stimulated to a lesser degree than IgG2a/b and was non-correlated with disease-burden. This would suggest the IgG1 is ineffective against bacterial clearance, either by acting as an anti-inflammatory agent or does not have protective functionality.

The functional role of IgA in *Burkholderia* infection has not been fully explored and therefore remains a gap in knowledge. While this and other studies have demonstrated increased antigen-specific IgA in vaccinated mice and correlated the increase with reduced disease, mechanistic functionality still has not been established. Here, nasal IgA levels were negatively correlated with inflammation and worse disease outcome, which suggests that nasal IgA prevented early colonization/infection, and subsequent inflammation induced by infection. Because inhalational melioidosis often leads to severe melioidosis, understanding the functional role of IgA in the respiratory tract, whether it be due to toxin neutralization, blocking adhesion, aggregating, complement activation, or neutrophil recruitment, may help to elucidate the heterogenous response in infected individuals.

### 4.3 IFN-γ and IL-10 axis in *Burkholderia* infection

IFN-γ has been studied as a correlate of protection for melioidosis, with the notion that it promotes cell-mediated immunity essential for killing the bacteria (39, 40). In this study, the levels of IFN-γ and other inflammatory Th1/17 cytokines were positively correlated with disease indicators, regardless of vaccination status, and follow a similar timeline to that of previously reported literature, particularly for IFN-γ (17–19). L1-vaccinated mice had elevated levels of inflammatory cytokines in response to infection, but were significantly decreased from FCDG-only control; with the reduction of disease indicators, this may suggest that reduced, but not ablated, inflammation could be beneficial. The stark decrease in Th17 cytokines in L1-vaccinated mice compared to FCDG-only mice may also decrease disease pathology since Th17 cytokines can promote tissue damage (41, 42). IL-17A and IL-22 in particular correlated the strongest with bacterial burden in lungs, therefore could be subjects for further investigation. Altogether, this and other studies have signified a fine balance between protective inflammation and a cytokine storm (5, 39, 43, 44). Positive correlations with macrophages and dendritic cells ratios indicate possible sources of inflammatory cytokines.

IL-10 is an anti-inflammatory cytokine that down-regulates inflammatory Th1-based cytokines and antigen-presentation, and promotes B cell proliferation amongst other functions (45). The exact mechanism(s) governing how IL-10 modulates the host-pathogen environment has not been studied, but there have been several studies linking it to severe melioidosis. Kessler et al. examined the role of IL-10 in *B. pseudomallei* infected PBMCs and found that neutralizing IL-10 increased IFN-γ levels, which has been documented as a protective cytokine against melioidosis (46). This relationship is further supported by the fact that diabetics, who have the leading risk factor for disease-state melioidosis, have an IL-10 skewed immunity (47).While this regulation can be beneficial for quelling runaway inflammation, in melioidosis it could impede bacterial clearance by inhibiting important cell-mediated cytokines like IFN-γ. We found IL-10 was significantly and uniformly increased in mice exhibiting a high-bacterial burden, suggesting that an increased-expression of IL-10 in the lung environment is correlated with worse disease indicators. When comparing levels of IL-10 in the lungs between control and L1-CRM_197_ + FCDG immunized mice, FCDG-only mice had significantly increased levels during infection, whereas L1-vaccinated mice maintained steady IL-10 levels, comparable to pre-infection levels. This would suggest that vaccination with the L1-peptide conjugate reduced production of IL-10; further studies could elucidate whether this reduction is antigen-dependent, for example diverting the immune reaction by eliciting IFN-γ, or a result of reduction of bacterial load through other mechanisms.

### 4.4 Th2 and mucosa stimulating cytokines in *Burkholderia* infection

IL-13 has a variety of functions that result in allergic response and IgE production, mucus hypersecretion, and/or tissue remodeling (48). In FCDG-immunized mice, IL-13 was moderately and negatively correlated with luminescent bacterial burden, suggesting that increased levels of IL-13 in naïve mice may be protective. Whether this is due to recruitment of allergy-based immune cells like eosinophils and mast cells, increased mucus production, or activation of anti-inflammatory M2 macrophages would still need to be investigated. Similar to IL-13, IL-9 is also involved in eosinophil and mast cell recruitment and stimulation, particularly in the lung environment when IL-13 is present (49), but also promotes cell proliferation, apoptosis inhibition (50), and regulatory T cell function (51). However, unlike IL-13, IL-9 correlated negatively with bacterial burden for both the immunization treatments, suggesting IL-9 plays a protective role in response, more so than IL-13. These results do not indicate if it is the same protective role in naïve mice vs antigen-immunized mice. Additionally, both IL-13 and IL-9 were not present at measurable levels in non-immunized mice, which suggests the nucleotide-based adjuvant FCDG may help prime and/or stimulate production of these cytokines.

There were several cytokines that have been described as being important to *B. pseudomallei* pathogenesis that we did not evaluate, including cytokines IL-8, IL-1β, IL-18, TGF-β (52–54). These cytokines are a part of the initial innate immune response in recruiting neutrophils, shown many times in literature to be vital for bacterial clearance (13, 55, 56). Our data indicated that neutrophils were not the largest myeloid cell population initially and increased in ratio as the course of infection progressed. This result could be due to a delay in neutrophil recruitment, or mice of both treatment groups that survived past the peak of infection were those that had increased levels of neutrophils.

### 4.5 A protective melioidosis vaccine will require balanced humoral and cell-mediated memory

**Figure 10:**
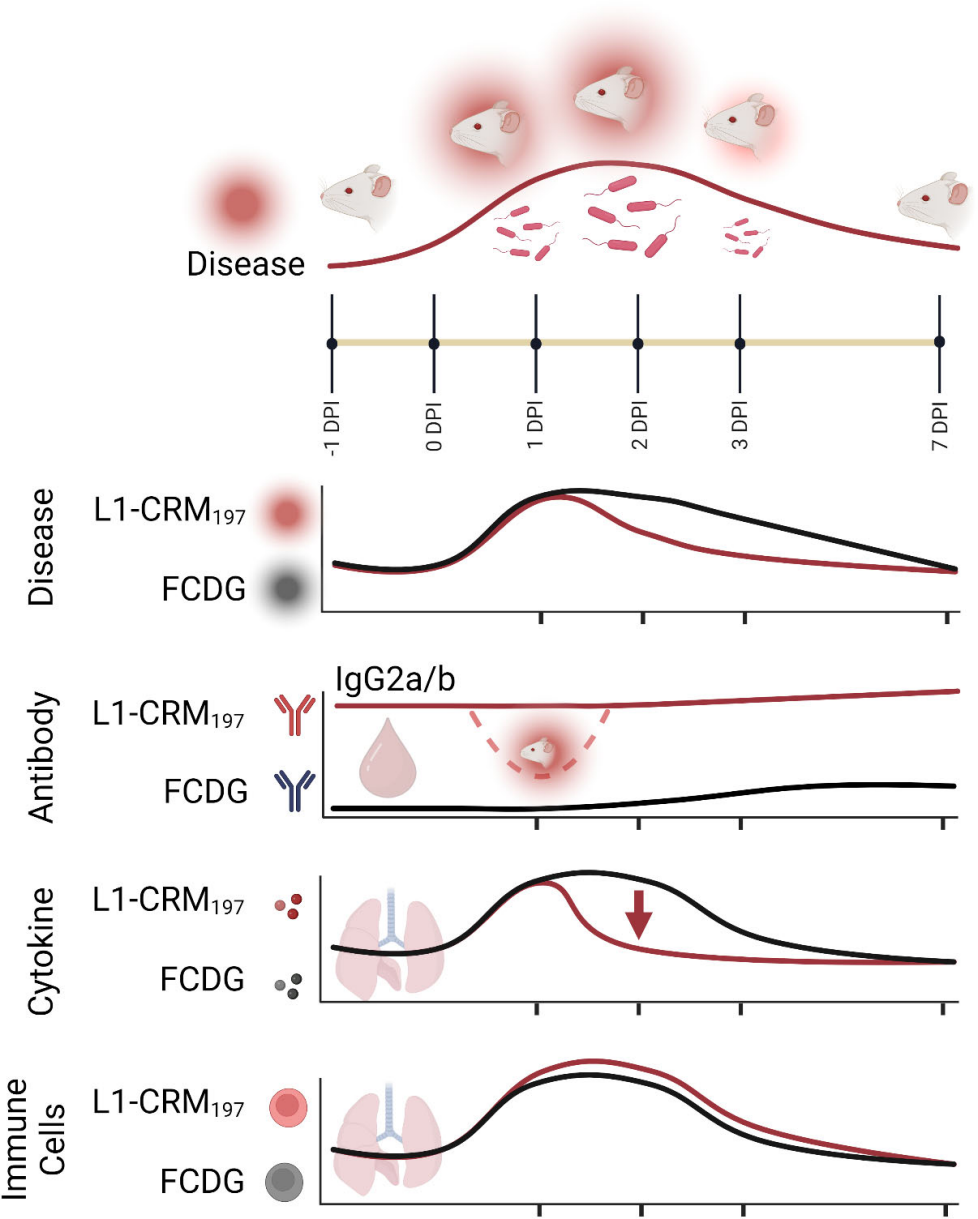
Summary model of intranasal vaccination with Bucl8-based peptide and bacterial challenge in outbred murine model of *Burkholderia* infection. Intranasal inoculation with (*B*) *thailandensis* of CD-1 mice immunized with a Bucl8-based peptide-conjugate led to decreased inflammation, bacterial burden, and promoted a Th1-driven antibody response. Infected/morbid mice are depicted by red radiance. Created with BioRender.com.

Prior literature has suggested that antibodies against different *Burkholderia* antigens can be protective, but a cell-mediated response may also be needed to clear infection (5, 57, 58). A combinatory approach that includes Th1 and Th2 stimulating elements may be needed to produce an effective subunit vaccine against *Burkholderia*. Here, we have shown that infection with a *B. thailandensis* in outbred CD-1 mice produces a primary disease course and immune response similar to other murine models of melioidosis. The vaccination study presented here is a comprehensive examination of individual immune responses, providing a moderate protective effect by the current formulation of the Bucl8-derived vaccine. We demonstrate this vaccine formulation alters the initial immune response to infection by eliciting antigen-specific antibodies, predominantly Th1-driven IgG2a/b, limiting weight loss and damaging inflammation at the height of infection, and decreasing bacterial burden (**Figure 10**). Contributions of the synthetic L1 peptide and CRM_197_-protein carrier alone to cell-mediated responses and protection will need additional characterization to fully understand the elicited immune response. Further alterations to the formula, like the adjuvant- or conjugate-type, could increase protection. Instead of on its own, L1-based antigen could be included into a subunit vaccine with other antigens to promote a stronger response.

## Data availability statement

The original contributions presented in the study are included in the article/**Supplementary Material**. Further inquiries can be directed to the corresponding author.

## Ethics statement

The animal study was reviewed and approved by WVU-IACUC protocol 1804013711.

## Author contributions

Conceptualization, writing, SL, MG. methodology/investigation, MG, SC. data curation, SL, MG, SC, LP. Statistical analysis, MG. supervision, project administration, SL All authors have read and agreed to the published version of the manuscript.
