# Supplementary material for: Intranasal immunization with a Bucl8-based vaccine ameliorates bacterial burden and pathological inflammation, and promotes an IgG2a/b dominant response in an outbred mouse model of Burkholderia infection
Source: Front Immunol. 2023 Jul 20;14:1177650. doi: 10.3389/fimmu.2023.1177650 (PMC10399622; doi:10.3389/fimmu.2023.1177650)
Supplement: Supplementary file 1 [file DataSheet_1.docx]

**Table S1.** Flow cytometry panels for lung cell characterization.

| Myeloid Panel | | | | | Lymphocyte panel | | | | | |
| --- | --- | --- | --- | --- | --- | --- | --- | --- | --- | --- |
| **Marker** | **Fluoro-phore** | **Source** | **Cat #** | **FACs Dilution** | **Marker** | **Fluoro-phore** | **Source** | **Cat #** | **FACs**  **Dilution** |  |
| CD11b | FITC | Biolegend | 101206 | 1:400 | CD11b | FITC | Biolegend | 101206 | 1:400 |  |
| CD11c | Percp/Cy5.5 | Biolegend | 117328 | 1:200 | B220 | APC | BD Pharmigen | 553089 | 1:400 |  |
| Ly6C | PE | Biolegend | 128008 | 1:800 | TCRβ | BV510 | Biolegend | 109233 | 1:200 |  |
| Ly6G | BV510 | Biolegend | 127633 | 1:200 | CD4 | BV421 | Biolegend | 100443 | 1:200 |  |
| F4/80 | APC | Biolegend | 123116 | 1:400 | CD8 | PE | Biolegend | 100708 | 1:800 |  |
| MHCII  (I-A/I-E) | BV421 | Biolegend | 107632 | 1:800 | Live/Dead | Near IR (876) | Invitrogen | 134982 | 1:200 |  |
| Live/Dead | Near IR (876) | Invitrogen | 134982 | 1:200 |  |  |  |  |  |  |

**Table S2.** Markers used to define cell populations.

| **Cell population** | **Markers** |
| --- | --- |
| Neutrophils | CD11b high, CD11c –, Ly6C low, Ly6G high |
| Inflammatory monocytes | CD11b high, CD11c –, Ly6C high, Ly6G low |
| Eosinophils | CD11b high, CD11c –, Ly6C low, Ly6G – |
| Macrophages | CD11b+, CD11c+, MHCII+ low, F4/80+ |
| Dendritic cells | CD11b+, CD11c+, MHCII+, F4/80- |

**
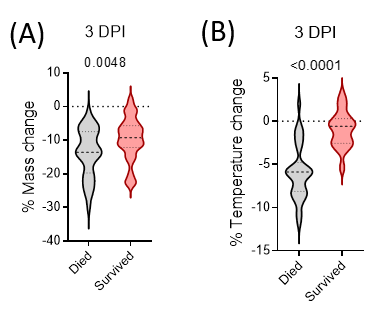
**

**Figure S1.** Percent change of weight and temperature at height of infection, 3 DPI. Both parameters were graphed and compared statistically between mice that succumbed to disease vs those that survived from all experiments (n _died_ =34, n _survived_ =55). Percent mass and temperature change were calculated by dividing a mouse’s end point or 3 DPI measurement, whichever came first, by the 0 DPI measurement. Student’s *t*-test. P values are shown.


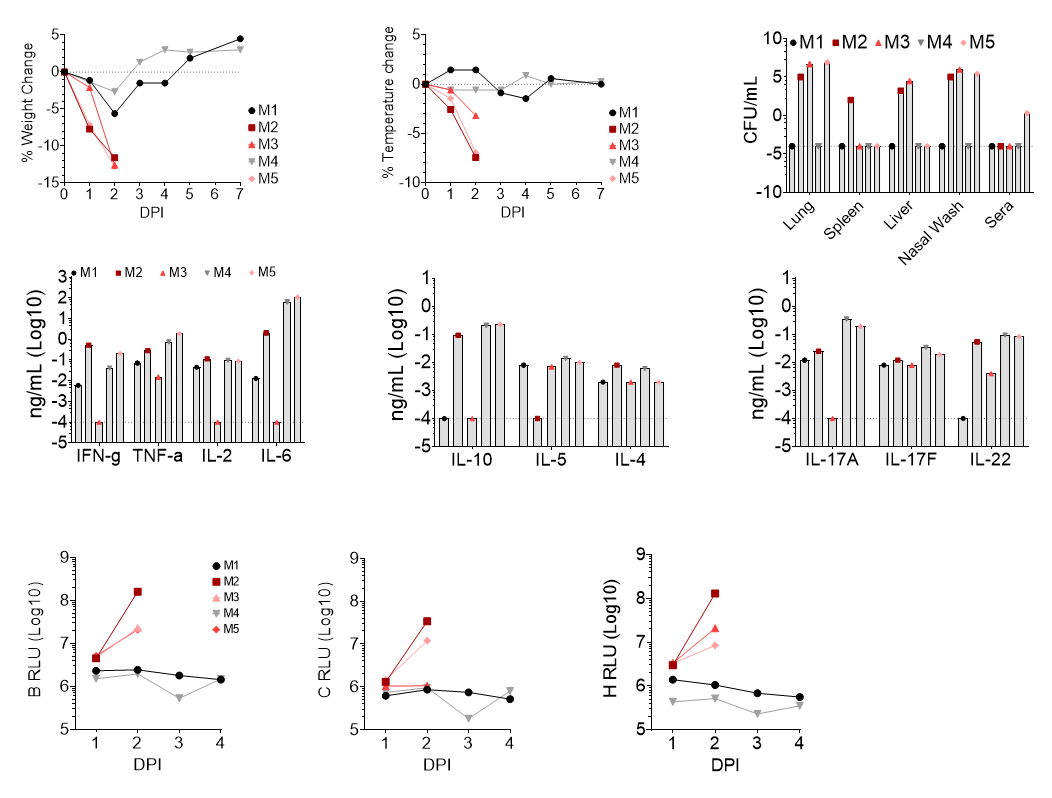
**Figure S2.** Corresponding graphs of Mouse #1-5 shown in Figure 3A. (**A**) Percent weight change and (**B**) temperature change of mice that were moribund 2 DPI (red) and mice that survived to 7 DPI (grey). (**C**) Bacterial loads from indicated tissues. (**D**) Cytokine levels of each mouse, separated by Th1, Th2, and Th17 cytokines. (**E**) Luminescent signal from body (B RLU), chest (C RLU), and head (H RLU).


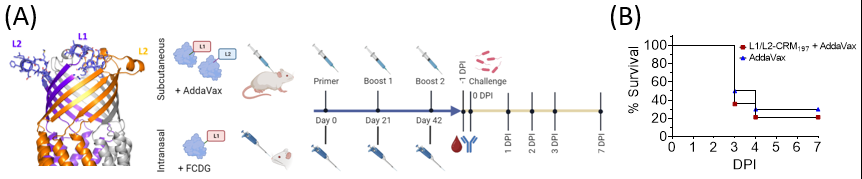


**Figure S3.** Evaluation of Bucl8-based subcutaneous vaccine following intranasal and inhalational bacterial challenge. (**A**) Experimental design. (*Left panel*) Cartoon representation of the outer-membrane-spanning β-barrel part of Bucl8 displaying surface-exposed loops L1 and L2 (adapted from Grund *et al*. 2021, *Vaccine (Basel)*). (*Right panel*) General schematic of vaccination timeline for both subcutaneous and intranasal immunization in subsequent figures. Blue arrow represents the immunization period and the yellow area the respiratory challenge. Created with BioRender.com. (**B**) Kaplan-Meier survival curves of female and male CD-1 mice subcutaneously immunized with L1/L2-CRM_197_ + AddaVax and challenged with 8 x 10^6^ CFU Bt E264. L1/L2 group n=14, 7 female/male; AddaVax n=10, 5 female/male. Two-way ANOVA with Šídák’s multiple comparison test. P values are shown. Day post infection; DPI.


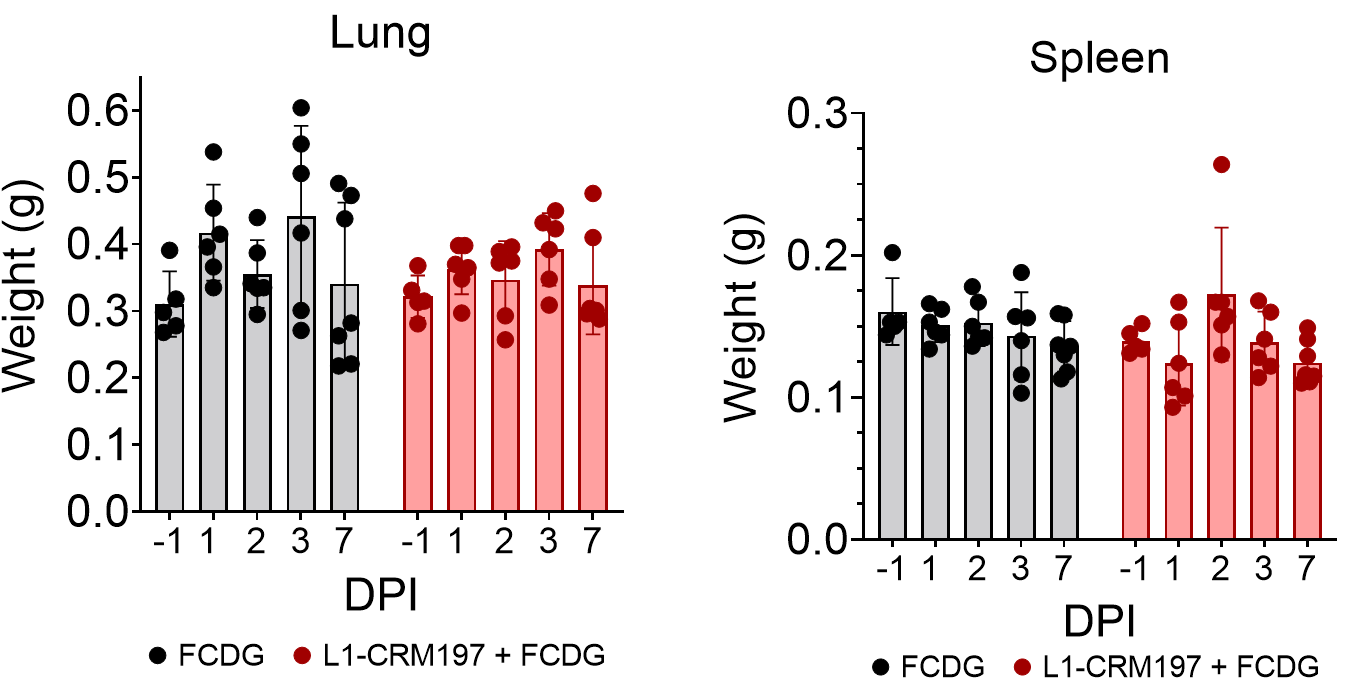


**Figure S4.** Wet weight of spleen and lung from Figure 5.


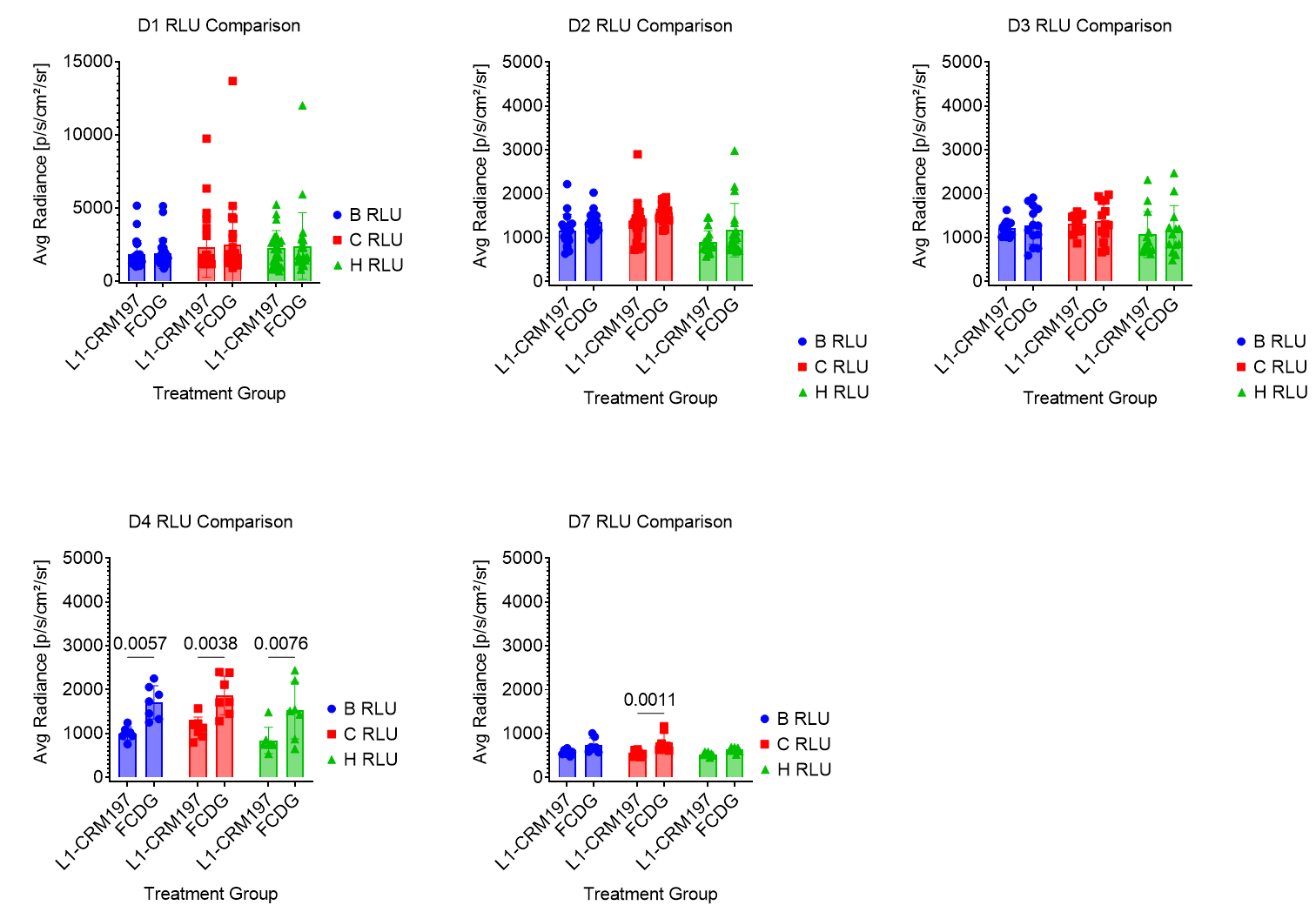


**Figure S5.** Luminescent signal of Bt E264-*lux* loads by DPI between L1-CRM_197_ and FCDG treatment groups. IVIS measurements were taken for the whole mouse body (B RLU), chest (C RLU) and head (H RLU) areas on 1-4, and 7 DPI. Student’s *t*-test. P values are shown. Corresponds to Figure 6 data.


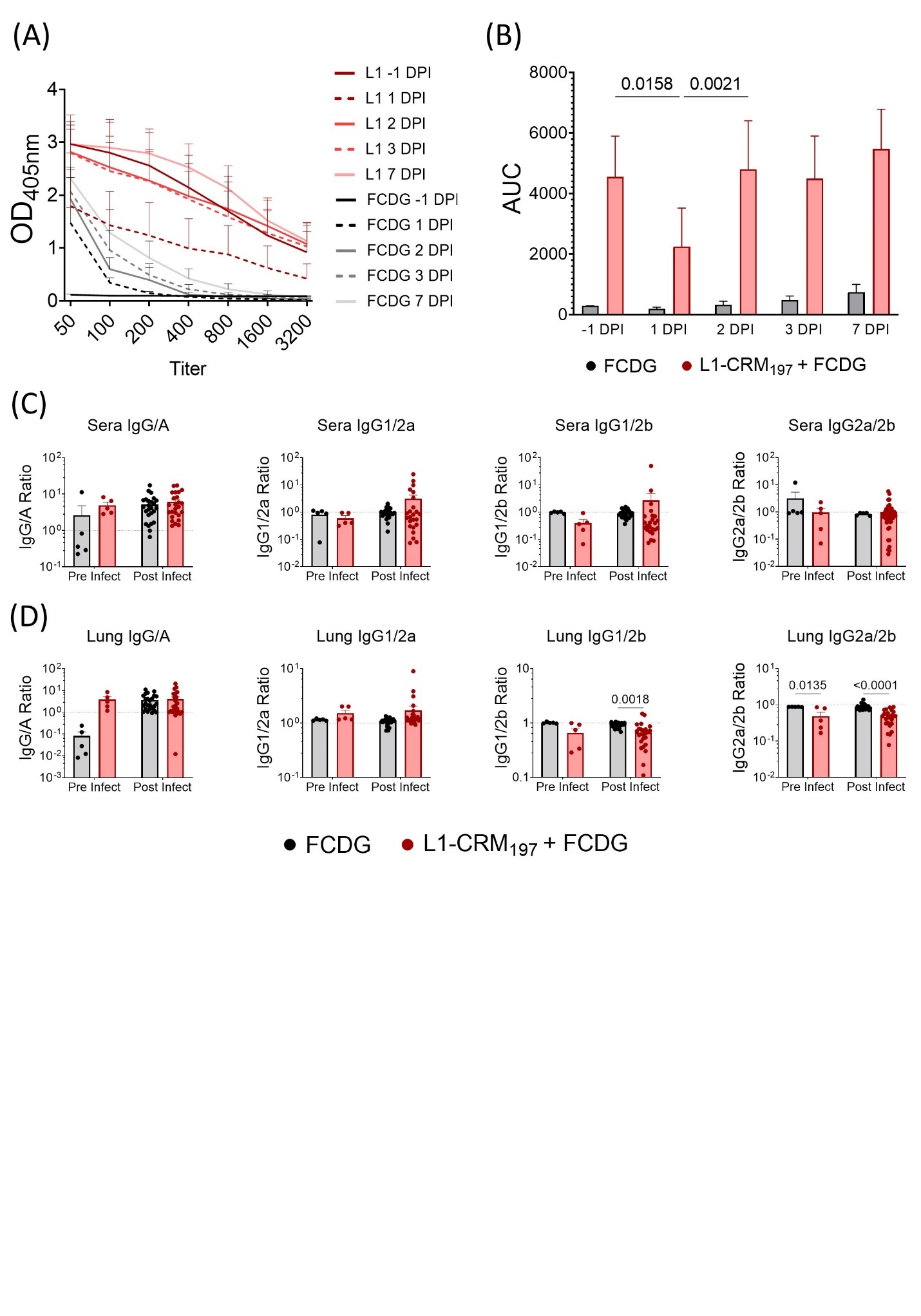


**Figure S6.** Sera IgG titer. Data corresponds to Figure 7A IgG graph. (A) Curves were generated by completing two-fold serial dilutions beginning at 1:50 and data separated by DPI group. SEM error bars. (B) Area under the curve (AUC) of panel A. Two-way ANOVA with Šídák’s multiple comparison test. DPI; days post infection. Ratios of antibody types and from Figure 6 were calculated for (**C**) serum and (**D**) lung supernatant. Two-way ANOVA with Šídák’s multiple comparison test. P values are shown.


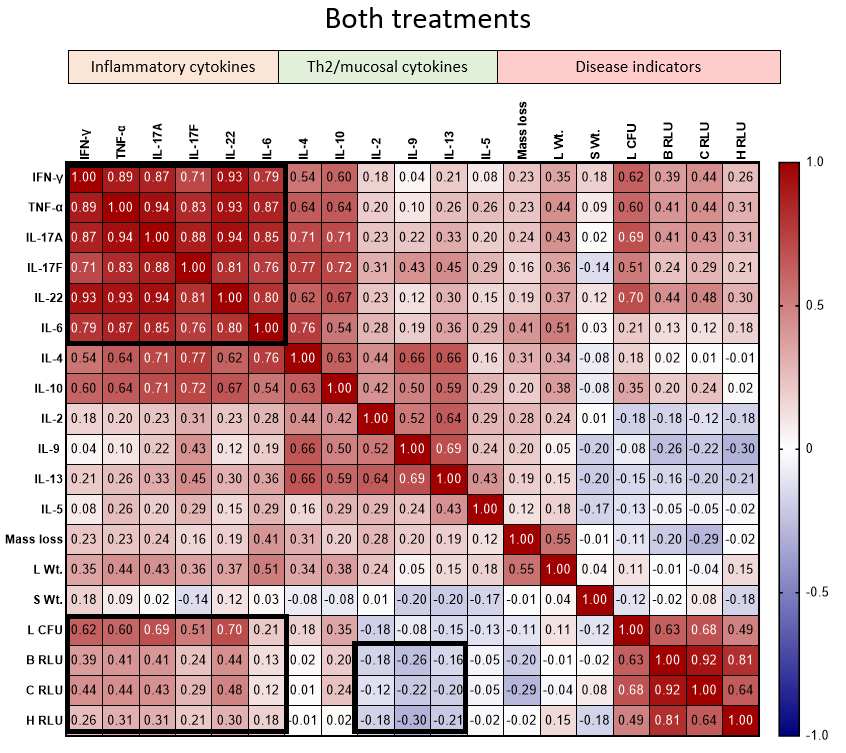


**Figure S7.** Spearman correlations of pulmonary levels of cytokines and disease indicators for L1-CRM_197_ and FCDG mice. L Wt; lung weight. S Wt; spleen weight.


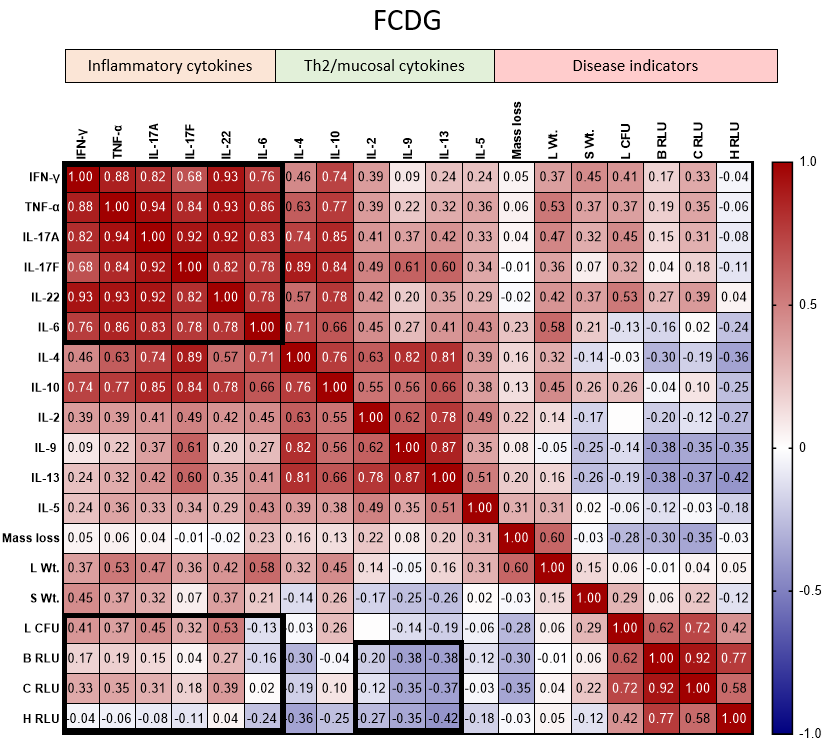


**Figure S8.** Spearman correlations of pulmonary levels of cytokines and disease indicators for FCDG mice. L Wt; lung weight. S Wt; spleen weight.


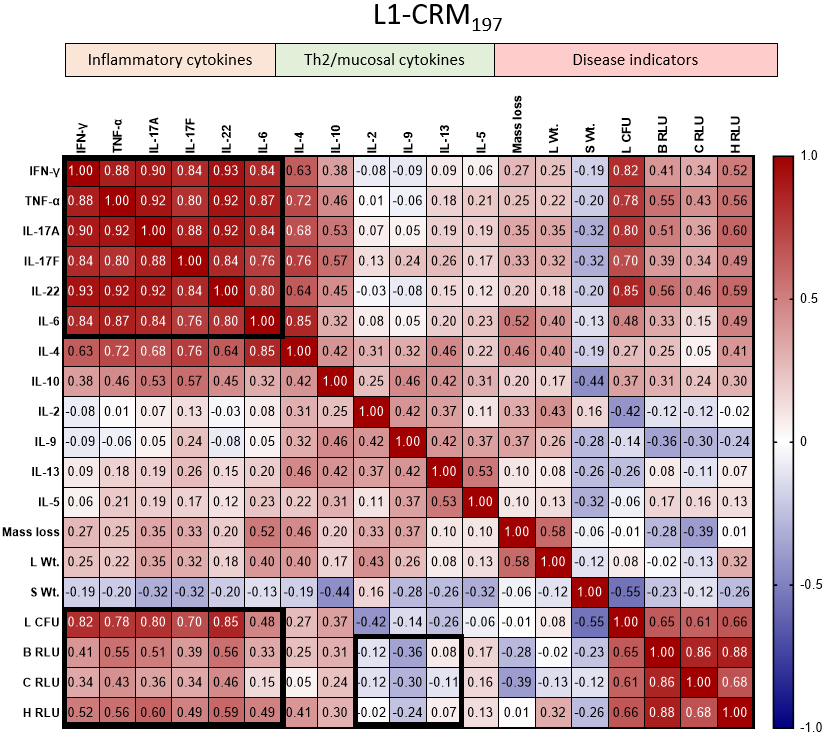


**Figure S9.** Spearman correlations of pulmonary levels of cytokines and disease indicators for L1-CRM_197_ mice. L Wt; lung weight. S Wt; spleen weight.


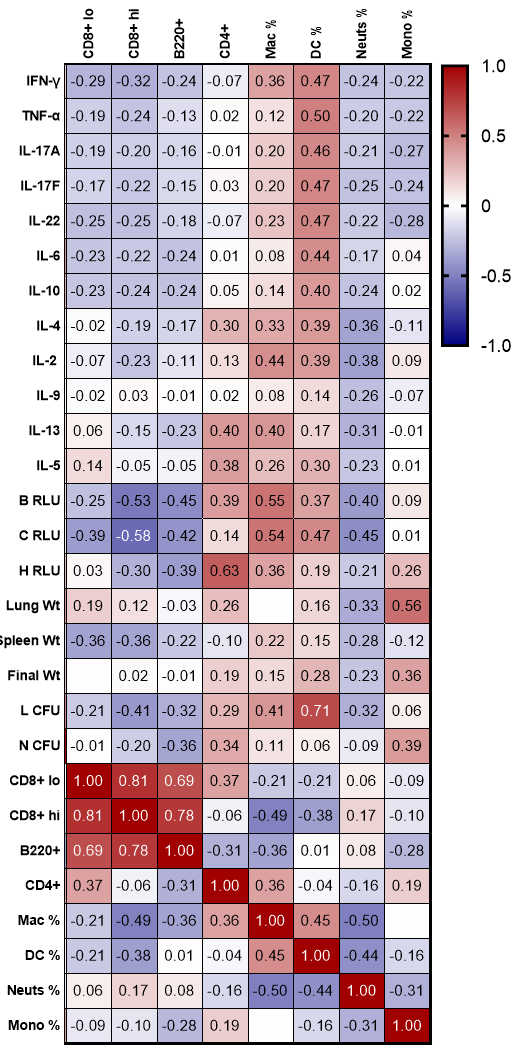


**Figure S10.** Spearman correlations of pulmonary levels of cytokines and cell populations for L1-CRM_197_ and FCDG mice. Wt; weight.
